# MANP: a novel particulate guanylyl cyclase A receptor/cGMP activator for resistant hypertension: preliminary first in human clinical trial results

**DOI:** 10.1186/2050-6511-16-S1-A3

**Published:** 2015-09-02

**Authors:** John C Burnett, Paul McKie, Denise Heublein, Brenda Huntley, Horng Chen, Valentina Cannone, Alessia Buglioni, Daniel McCormick

**Affiliations:** 1 Cardiorenal Research Laboratory, Division of Cardiovascular Diseases, and Department of Biochemistry and Molecular Biology, Mayo Clinic, Rochester, MN 55905, USA

## Introduction

MANP is a novel natriuretic peptide developed at the Mayo Clinic following its discovery from a familial frameshift mutation of the atrial natriuretic peptide (ANP) gene (NPPA) [1,2]. MANP possesses the 28 amino acid (AA) core structure of ANP with a novel 12 AA extended C-terminus resulting in a 40 AA peptide. MANP activates the particulate guanylyl cyclase A (pGC-A) receptor with equal potency to ANP and is highly resistant to degradation by neprilysin. In vivo, MANP has greater and longer lasting blood pressure (BP) lowering, natriuretic and aldosterone suppressing actions than ANP [3]. In animal models of hypertension, MANP potently reduces BP and enhances renal function while in experimental hypertensive heart failure, MANP has more potent renal and aldosterone suppressing actions than the cyclic guanylyl monophosphate (cGMP)-activating therapeutic agent nitroglycerin [4]. In vivo administered subcutaneously, MANP activates plasma cGMP 10-fold greater than ANP with a 4-fold greater half-life [3,4]. Based upon its potent cGMP mediated cardiorenal actions and studies which suggest that human hypertension may represent a relative natriuretic peptide deficiency state, MANP is being developed for treatment of severe resistant hypertension.

## Methods and results

MANP (ZD100, Zumbro Discovery) recently entered a first in human trial in patients with hypertension. A Phase 1 (Part A) single ascending dose (SAD) study was recently completed. One dose of MANP was administered to hypertensive patients in whom anti-hypertensives were stopped for two weeks prior to MANP administration. Three cohorts of 4 subjects were studied (1, 2.5 and 5 ug/kg subcutaneous injection) with dose escalation stopped if more than two subjects had greater than 30 mmHg reduction in systolic BP. Endpoints were safety, BP, pharmacokinetics, renal actions and neurohumoral function. Initial BP and safety data will be presented for the first time.

## Conclusion

MANP is a novel, sustained-acting pGC/cGMP activating peptide, which goes beyond ANP in reducing BP, augmenting renal function and suppressing aldosterone. BP and safety data will be presented for the first time from the recently completed first in human SAD study in patients with hypertension.
